# A phase I/II randomized, double-blinded, placebo-controlled trial of a self-amplifying Covid-19 mRNA vaccine

**DOI:** 10.1038/s41541-022-00590-x

**Published:** 2022-12-13

**Authors:** Jenny G. Low, Ruklanthi de Alwis, Shiwei Chen, Shirin Kalimuddin, Yan Shan Leong, Tania Ken Lin Mah, Natalene Yuen, Hwee Cheng Tan, Summer L. Zhang, Jean X. Y. Sim, Yvonne F. Z. Chan, Ayesa Syenina, Jia Xin Yee, Eugenia Z. Ong, Rose Sekulovich, Brian B. Sullivan, Kelly Lindert, Sean M. Sullivan, Pad Chivukula, Steven G. Hughes, Eng Eong Ooi

**Affiliations:** 1 Programme in Emerging Infectious Diseases, Duke-National University of Singapore Medical School, 8 College Road, 169857 Singapore, Singapore; 2 Department of Infectious Diseases, Singapore General Hospital, 169608 Singapore, Singapore; 3 Viral Research and Experimental Medicine Center, SingHealth Duke-NUS Academic Medical Center, Singapore, Singapore; 4 Department of Biological Sciences, Nanyang Technological University, 639798 Singapore, Singapore; 5 Arcturus Therapeutics Inc, San Diego, CA 92121 USA

**Keywords:** Drug development, RNA vaccines

## Abstract

Coronavirus disease-19 (Covid-19) pandemic have demonstrated the importantance of vaccines in disease prevention. Self-amplifying mRNA vaccines could be another option for disease prevention if demonstrated to be safe and immunogenic. Phase 1 of this randomized, double-blinded, placebo-controlled trial (*N* = 42) assessed the safety, tolerability, and immunogenicity in healthy young and older adults of ascending levels of one-dose ARCT-021, a self-amplifying mRNA vaccine against Covid-19. Phase 2 (*N* = 64) tested two-doses of ARCT-021 given 28 days apart. During phase 1, ARCT-021 was well tolerated up to one 7.5 μg dose and two 5.0 μg doses. Local solicited AEs, namely injection-site pain and tenderness were more common in ARCT-021vaccinated, while systemic solicited AEs, mainly fatigue, headache and myalgia were reported in 62.8% and 46.4% of ARCT-021 and placebo recipients, respectively. Seroconversion rate for anti-S IgG was 100% in all cohorts, except for the 1 μg one-dose in younger adults and the 7.5 μg one-dose in older adults. Anti-S IgG and neutralizing antibody titers showed a general increase with increasing dose, and overlapped with titers in Covid-19 convalescent patients. T-cell responses were also observed in response to stimulation with S-protein peptides. Taken collectively, ARCT-021 is immunogenic and has favorable safety profile for further development.

## Introduction

The coronavirus disease 2019 (Covid-19) pandemic, caused by the severe acute respiratory syndrome coronavirus-2 (SARS-CoV-2), has devastated lives globally^1^. Accelerated vaccine deployment has started to reverse this trend^2,3^. The most effective vaccines have been mRNA vaccines^4–6^. An alternative synthetic mRNA platform—self-amplifying RNA (sa-mRNA)—may have the advantage of potentially allowing smaller doses to be administered^7,8^. However, data on the safety and immunogenicity profile of sa-mRNA vaccines remain limited.

Here we report our findings of a first-in-human clinical trial of the safety, tolerability and immunogenicity of ARCT-021, a self-transcribing and replicating mRNA (STARR^TM^) vaccine candidate for the prevention of Covid-19. The mRNA is a replicon that comprises the Venezuelan equine encephalitis virus (VEEV) genome in which the structural genes have been replaced with the SARS-CoV-2 full-length spike (S) gene, and formulated with the proprietary LUNAR^®^ lipid nanoparticle (LNP). Translation of the replicon produces a multi-protein replicase complex that amplifies a subgenomic mRNA for elevated expression of the S glycoprotein. Preclinical experiments showed that a single dose of ARCT-021 elicited strong Th1-predominant humoral and cellular immune responses against the S protein that protected K-18 human ACE2 transgenic mice from lethal SARS-CoV-2 challenge^9^.

## Results

### Trial participants

**Fig. 1:**
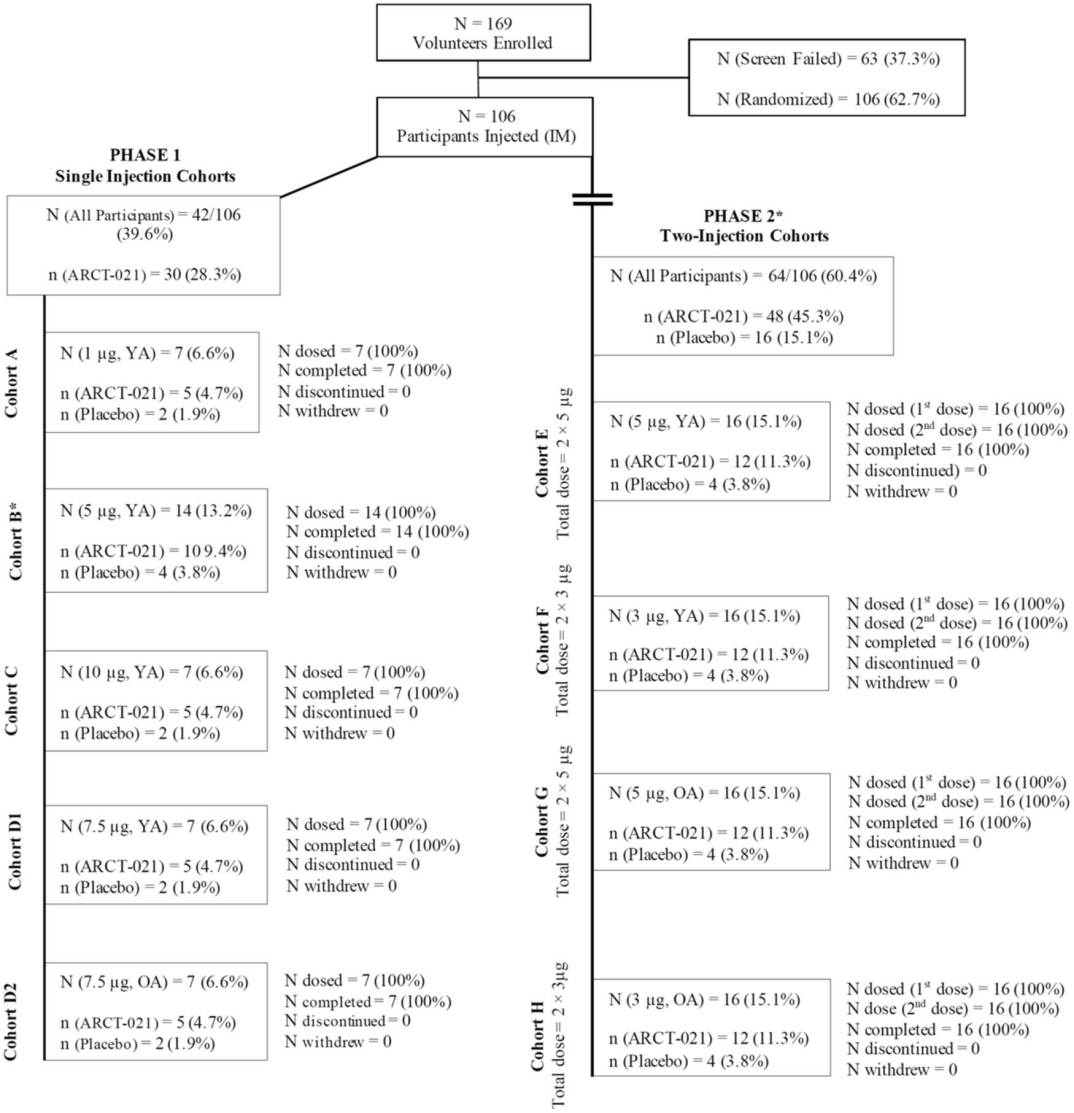
Enrollment and randomization of trial participants. N number of participants in the cohort, n number of participants in each treatment group, OA older adults (56–80 years), YA younger adults (21–55 years). Phase 1—Single injection, escalating dose. Study duration 56 days post first injection. Phase 2—Two same dose injections, 28 days apart. Study duration 85 days post first injection.

In total, 169 healthy volunteers were screened, with 106 participants randomized and injected. The distribution of participants by cohort is shown in Fig. 1. All participants received assigned doses of ARCT-021. All enrolled participants completed the planned study scheduled trial visits. A summary of demographic characteristics by treatment assignment is presented in Table 1.

**Table 1:** Demographic characteristics of the participants, according to dose cohort and age group^a^.

Cohort	Single-dose cohorts							Two-dose cohorts					
	Age 21–55					Age 56–80		Age 21–55			Age 56–80		
Variable	Cohort A 1 µg	Cohort B 5 µg	Cohort C 10 µg	Cohort D1 7.5 µg	Pooled placebo cohorts A–D1	Cohort D2 7.5 µg	Cohort D2 Placebo	Cohort E 5 µg	Cohort F 3 µg	Pooled placebo cohorts E–F	Cohort G 5 µg	Cohort H 3 µg	Pooled placebo cohorts G–H
Number of participants	5	10	5	5	10	5	2	12	12	8	12	12	8
Sex—*n* (%)													
Male	3 (60)	6 (60)	2 (40)	4 (80)	8 (20)	5 (100)	1 (50)	8 (67)	10 (83)	5 (63)	11 (92)	8 (67)	7 (88)
Female	2 (40)	4 (40)	3 (60)	1 (20)	2 (20)	0 (0)	1 (50)	4 (33)	2 (17)	3 (38)	1 (8)	4 (33)	1 (13)
Race—*n* (%)^b^													
Asian	5 (100)	10 (100)	5 (100)	4 (80)	10 (100)	5 (100)	2 (100)	11 (92)	10 (83)	7 (88)	12 (100)	12 (100)	7 (88)
White	0 (0)	0 (0)	0 (0)	1 (20)	0 (0)	0 (0)	0 (0)	0 (0)	1 (8)	1 (13)	0 (0)	0 (0)	1 (13)
American Indian or Alaskan Native	0 (0)	0 (0)	0 (0)	0 (0)	0 (0)	0 (0)	0 (0)	1 (8)	0 (0)	0 (0)	0 (0)	0 (0)	0 (0)
Other	0 (0)	0 (0)	0 (0)	0 (0)	0 (0)	0 (0)	0 (0)	0 (0)	1 (8)	0 (0)	0 (0)	0 (0)	0 (0)
Hispanic Ethnic Group—*n* (%)^b^	0 (0)	0 (0)	0 (0)	0 (0)	0 (0)	0 (0)	0 (0)	0 (0)	0 (0)	0 (0)	0 (0)	0 (0)	0 (0)
Age (years)^c^													
Mean (SD)	35 (9.17)	39.5 (7.15)	41.4 (12.48)	40.4 (10.41)	33.5 (8.72)	64 (5.61)	61.5 (6.36)	34.5 (8.33)	38 (9.34)	39.1 (7.95)	62.3 (4.63)	61.7 (3.77)	63.3 (5.70)
Median [range]	32.0 [28–50]	40.0 [29–51]	44.0 [24–55]	47.0 [24–48]	34.5 [21–46]	64.0 [57–70]	61.5 [57–66]	35.5 [21–47]	35.0 [22–51]	37.0 [30–51]	62.0 [56–70]	61.5 [56–68]	64.5 [56–71]

*N* total number of subjects in each dose cohort, *n* number of subjects contributing to the summary, *SD* standard deviation.

^a^Percentages may not total 100 because of rounding.

^b^Race and ethnic group were reported by the participant.

^c^The age of the participants was the age at the time of screening.

### Safety

**Fig. 2:**
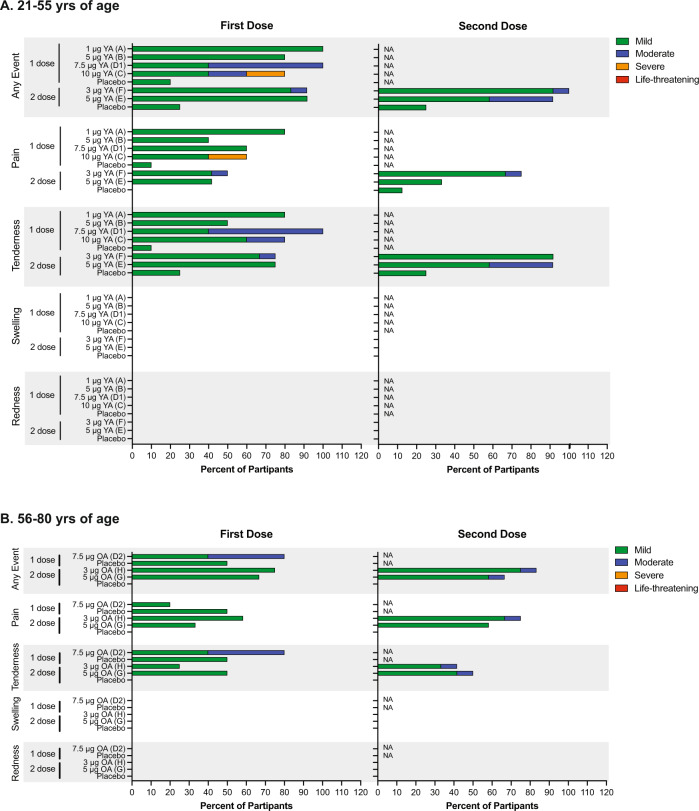
Local solicited adverse events. Shown are the percentages of vaccinated participants reporting local solicited events. Data are represented for age groups, 21–55 years (**A**) and 56–80 years (**B**). Severity of local solicited Events are displayed as mild (green), moderate (blue), severe (orange), or life-threatening (red). *Note, injection-site swelling and injection-site redness were not reported. YA younger adults (21–55), OA older adults (56–80).

**Fig. 3:**
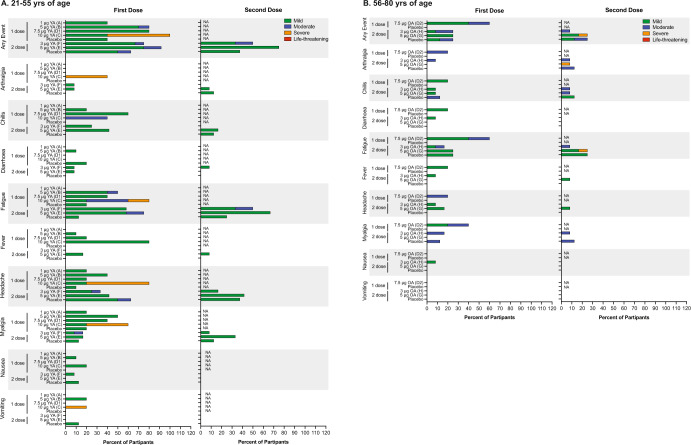
Systemic solicited adverse events. Percentage of systemic solicited events reported in vaccinated participants aged 21–55 years (**A**) and 56–80 years (**B**). Severity of systemic solicited Events are displayed as mild (green), moderate (blue), severe (orange), or life-threatening (red). YA younger adults (21–55), OA older adults (56–80).

A summary of solicited local and systemic adverse events (AE) is presented in Figs. 2 and 3. ARCT-021 was generally well tolerated up to the 7.5 μg dose. The 10 μg dose was associated with more local and systemic solicited AE, including grade 3 severity. Although the study stopping rules were not met, the protocol was amended to remove further dose escalation. A younger (Cohort D1) and older adult (Cohort D2) one-dose cohorts at the 7.5 μg dose level were added following safety review. A single serious adverse event due to cellulitis of the foot in a placebo recipient following an insect-bite was reported. It was medically attended and resolved after 2 days. All participants in the two-dose cohorts completed the vaccinations without any delay.

### Solicited treatment emergent adverse events—local reactions

Sixty-nine (88.5%) participants who received ARCT-021 reported local solicited AEs, as compared to 21.4% of placebo-treated participants (Fig. 2). These included injection-site tenderness and pain in 73.1% and 60.3% of participants, respectively. In the two-dose younger adult cohorts, local solicited AE occurred in 11(91.7%) following the first dose at both 5.0 and 3.0 μg and in 11 (91.7%) and 12 (100%), respectively, following the second dose. In older adults, local solicited events occurred in 8 (66.7%) and 9 (75%) following the first dose and in 8 (66.7%) and 10 (83.3%) following the second dose in the 5.0 and 3.0 μg dose cohorts, respectively. A higher proportion of participants experienced moderate grade injection-site tenderness with the second dose. At the same dose level, a higher proportion of younger than older adults experienced ≥grade 2 solicited AE. There was no reported injection-site erythema or swelling. There was one grade 3 injection-site pain at the 10μg dose. There were no grade 4 local reactions and no discernable dose relationship.

### Solicited treatment emergent adverse events—systemic reactions

Overall, 62.8% of participants who received ARCT-021 reported a systemic solicited AE as compared to 46.4% amongst the placebo recipients (Fig. 3). The most common events were fatigue (50.0%), headache (34.6%), myalgia (28.2%), chills (25.6%), and fever (14.1%) and were more common in younger than older adults. Systemic AEs were primarily mild or moderate with no grade 4 events reported. Grade 3 solicited events occurred primarily in the 10 μg dose cohort. Two grade 3 events (fatigue and arthralgia) occurred in a single participant following the second dose in the 5.0 μg older adult cohort. Severity of systemic AEs trended with dose level. Most systemic AEs commenced within 1–2 days after vaccination and resolved or reduced in severity within 7 days.

### Unsolicited systemic treatment emergent adverse events

Unsolicited AEs are summarized in Supplementary Table 1. In general, more participants vaccinated with ARCT-021 reported unsolicited AEs after vaccination. Five were severe, occurring primarily in the 10 μg dose cohort. These events occurred on the day after injection and resolved or improved in severity within 2 days after onset. One participant in older adult 3.0 μg two-dose cohort (F) experienced grade 3 transaminitis 57 days after the last dose of ARCT-021. This event was classed as unlikely related by the study investigator and resolved after 18 days. No severe events occurred in placebo-treated participants.

### Abnormal hematological and biochemical findings

There appeared to be a dose-related trend for ≥grade 2 lymphopenia with 0%, 25%, 26.5%, 30.0%, and 40.0% of participants affected at the 1.0, 3.0, 5.0, 7.5, and 10 μg dose levels, respectively. Onset of lymphopenia occurred within 24 h after injection and resolved uneventfully, generally within a day. The incidence was similar following the first (14 of 78 participants [17.9%]) and second dose (10 of 48 participants [20.8%]). Three of 48 (6.3%) participants had ≥grade 2 lymphopenia following both the first and second injection. There was no trend for increased severity following the second injection. The incidence was similar in older (24.1%) and younger adults (26.5%). There were 10 (12.8%) Grade 2 neutropenia events, the incidence of which was higher in older (17.2%) than younger (10.2%) adults. All neutropenic episodes were asymptomatic, resolved spontaneously and did not result in any sequelae.

Post-baseline elevations of ALT occurred in 5 ARCT-021 treated participants (Supplementary Table 3). Two of these participants had ALT elevation at screening, which had normalized at day 1. No elevations of hepatic enzymes were observed in placebo participants. There were no other notable laboratory abnormalities.

### SARS-CoV-2-binding antibodies and neutralization response

**Fig. 4:**
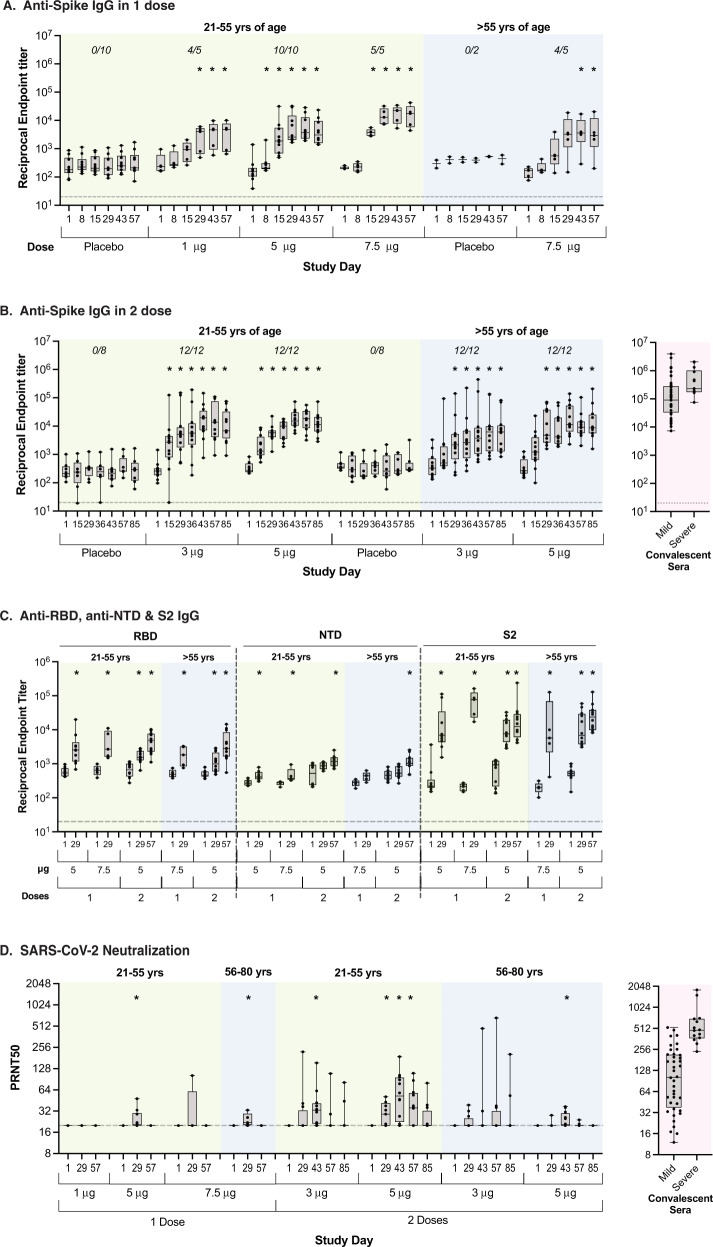
SARS-CoV-2 specific antibody responses. Shown are IgG reciprocal endpoint titers to SARS-CoV-2 whole ectodomain Spike using Luminex immune-Assay following 1 dose (**A**) and 2 doses (**B**) of vaccines. IgG reciprocal titers to the Spike subdomains, i.e., receptor-binding domain (RBD), N-terminal binding domain (NTD), and domain 2 (S2) using Luminex immuno-assay is represented for selected cohorts and timepoints (**C**). Antibody neutralization of live SARS-CoV-2 virus was measured using a BSL3 plaque reduction neutralization assay (PRNT) and represented as 50% neutralization titers (PRNT50) (**D**). Data are presented as boxplots with median and interquartile range (IQR). For each cohort, antibody data from each timepoint were compared to baseline (i.e., day 1) using Mann–Whitney U test (all *P*-values < 0.05 are marked with asterisk (*)). Each dot represents an individual participant. IgG seroconversion rates at day 29 for 1 dose cohorts and day 57 post-vaccination for 2 dose cohorts are specified on panels **A** and **B**, respectively.

Seroconversion rate for anti-S IgG was 100% in all cohorts except for the one-dose younger adult cohort A (1 μg) and the one-dose older adult cohort D2 (7.5 μg), which were both 80%. The IgG response increased with increasing dose (Fig. 4A, Table S5, S6, S7, and S8), although there was a trend for better responses at the 5.0 μg dose than at the 7.5 μg dose in older adults after a single dose (compared at day 29 in the two-dose cohorts). With the exception of the 1 μg dose, S-binding IgG titers overlapped with the range of antibodies in Covid-19 convalescent plasma (Fig. 4B). Vaccine-induced IgG antibodies bound the various S-protein subdomains, including the RBD, NTD and the highly conserved S2 (Fig. 4C). PRNT_50_ titers increased with increasing dose, while the responses were similar following one-dose of 5.0 μg and 7.5 μg. At doses ≥3 μg, PRNT_50_ titers were within the range observed in convalescent sera from patients with mild to moderate Covid-19 (Fig. 4D).

### SARS-CoV-2 T-cell response

**Fig. 5:**
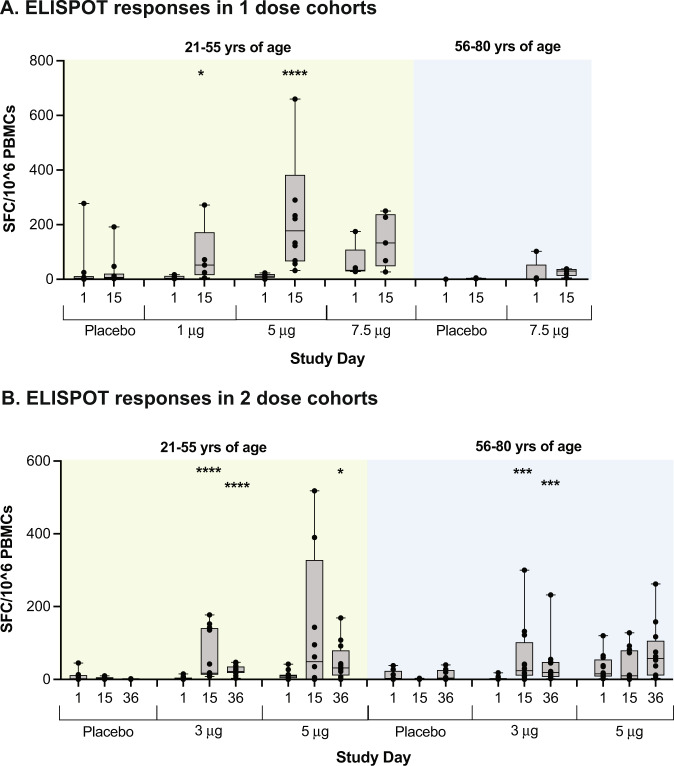
SARS-CoV-2 specific T-cell responses to ARCT-021. T-cell responses were measured using an interferon gamma (IFNγ) enzyme-linked immune absorbent spot (ELISPOT) assay and represented as spot-forming cells (SFC) per million PBMCs (**A** and **B**). Displayed are cumulative ELISPOT results for four peptide pools spanning the SARS-CoV-2 Spike. The data are represented as box and whisker plots where the middle horizontal line marks the median. Each dot represents an individual participant. Within each cohort, antibody data from each timepoint were compared to baseline (i.e., day 1) using Mann–Whitney *U* test (*P*-values marked with *0.01 < *P* < 0.05, **0.001 < *P* < 0.01, ***0.001 < *P* < 0.01, ****P < 0.0001).

T-cell responses were observed on both ELISPOT and ICS in response to stimulation with six peptide pools covering the entire S glycoprotein (Fig. S1). IFNγ ELISPOT responses were generally maximal at day 15, and were similar at the 5.0 and 7.5 μg dose in younger adults (Fig. 5A and Fig. S2). Responses in the 5.0 μg older adult cohort peaked after the second dose (Fig. 5B and Fig. S3). IFNγ ELISPOT responses were generally higher in younger adults (Fig. 5, Fig. S2, and Fig. S3). CD4 T-cell IFN-γ responses as measured by ICS showed the greatest values at day 29 in the 1.0 μg and 5.0 μg single-dose cohorts (Fig. S5). CD4 T-cell IFN-γ responses were generally greater in older adults than younger adults at the 5.0 and 7.5 μg doses and were similar between the two age groups at the 3 μg dose. A further increase in CD4 T-cell IFN-γ responses following second dose was only seen in the 5.0 μg younger adults. At all tested doses, the CD4 T-cell responses were Th1 dominant (Fig. S6). The CD8 T-cell IFN-γ cytokine responses by ICS showed the highest values at day 29 in the 5.0 μg single-dose cohort. CD8 IFN-γ responses in the older adult cohorts were higher than the responses observed in the younger adult cohorts at the same dose (Fig. S4). There was no further increase in CD8 T-cell IFN-γ responses following the second dose (Fig. S4).

## Discussion

Covid-19 will likely become a globally endemic disease, with new SARS-CoV-2 variants of concern emerging stochastically. New vaccines may thus be needed periodically to prevent those variants that effectively escape immunity afforded by current vaccines and SARS-CoV-2 variant infections. We found ARCT-021 to have an overall favorable safety profile up to 7.5 ug dose. Majority of the AEs were either mild or moderate. Transient lymphopenia and neutropenia, possibly due to innate immune driven redistribution of lymphocytes^10^ were observed, similar to other mRNA vaccines^11,12^.

ARCT-021 is also immunogenic. Anti-S IgG antibody levels were within the range of convalescent plasma after the first dose. Anti-S antibody of different types—IgM and IgA—in addition to IgG were detected. These antibodies can elicit Fc-mediated functions, such as complement activation, antibody-dependent cellular cytotoxicity (ADCC) and phagocytosis. Indeed, onset of mRNA vaccine-mediated protection against Covid-19 was associated with the development of anti-S antibodies that showed ADCC activity, along with S-reactive T cells, even before the development of neutralizing antibodies^13,14^. These findings collectively indicate that other functions of antibodies besides virus neutralization play key roles in protecting against Covid-19^14,15^.

Although a single dose of ARCT-021 was immunogenic, the second dose did not boost antibody titers dramatically. Moreover, the SARS-CoV-2 neutralizing antibody titers were low. It has to be noted that we have not modified (i.e., prestabilized or mutated the furin site) the S gene sequence in ARCT-02 but have kept it the same as that which was shown to be both immunogenic and efficacious in our preclinical study^9^. This may have an effect in modulating the immunogenicity of ARCT-021 in humans. Indeed, a modified S gene sa-mRNA construct (ARCT-154) has successfully progressed through immunogenicity trials to phase III clinical trial in Vietnam, which showed 95.3% efficacy against severe Covid-19 (Clinicaltrials.gov identifier: NCT04480957) (https://ir.arcturusrx.com/news-releases/news-release-details/arcturus-announces-self-amplifying-covid-19-mrna-vaccine).

Besides antibodies, ARCT-021 also elicited T-cell responses against overlapping peptide pools of the S protein. Notably, the T-cell responses were more Th1 than Th2 in phenotype. The body of evidence that T cells are important to immunity, even in the absence of neutralizing antibodies, is now sizeable^13^. We have shown in preclinical studies on ARCT-021 that vaccine-induced protection against SARS-CoV-2 was mediated primarily by CD8 T cells rather than B cells^9^. In NHPs, cellular immunity compensated for low antibody levels in protecting against SARS-CoV-2 infection^16^. Clinically, delayed T- but not B-cell responses were associated with severe disease^17^. In healthcare workers, the presence of CD8 T cells that recognized the nsp12 of SARS-CoV-2 aborted SARS-CoV-2 infection, even to the extent of abrogating seroconversion. Finally, recognition of epitopes spanning the S protein by CD8 T cells could also protect against neutralizing antibody-evading variants of concern (VOC)^18^, which may be particularly important for resilience against future VOCs^18–21^.

A striking observation in the course of the development of a sa-mRNA vaccine against Covid-19 is the difference in immunogenicity in mice compared to humans; both the preclinical study and ARCT-021 clinical trial tested unmodified S gene in the sa-mRNA construct. Similar findings were made with another candidate sa-mRNA vaccine against Covid-19^22,23^. Although the biological basis for this difference remains uncertain, ancillary investigations showed that the innate immune response to ARCT-021 was comparable to those elicited by other successful live vector vaccines (Ong et al, manuscript submitted), suggesting the potential for further development.

Taken in totality, our findings show that sa-mRNA vaccine is immunogenic and has a favorable safety profiles. Further development is warranted to realize its full potential.

## Methods

### Trial design, participants, and study approval

ARCT-021-01 is a randomized, double-blinded, placebo (0.9% saline) controlled study to assess the safety, tolerability and immunogenicity of different dose levels of ARCT-021. The primary endpoint was safety and tolerability; secondary and exploratory endpoints were antibody and T-cell responses. The trial was conducted at the Singapore Health Services (SingHealth) Investigational Medicine Unit, following approvals by the SingHealth Centralized Institutional Review Board (CIRB F/2020/2553) and the Singapore Health Sciences Authority. The trial was registered in clinicaltrial.gov (NCT04480957). A safety review committee reviewed data regarding safety and overall trial progress, including dose escalation decisions.

The trial comprised two overlapping parts and evaluated a range of doses of ARCT-021 versus placebo given as one- or two-dose administration to younger (21–55 years) and older (56–80 years) adult participants. In the Phase 1 part, a one-dose administration (dose levels 1.0, 5.0, 7.5, and 10 μg ARCT-021 versus placebo) was given as an intramuscular (IM) injection to younger adults (Cohorts A, B, D1, and C, respectively) and a single dose (7.5 μg ARCT-021 versus placebo) was given to older adults (Cohort D2). In the Phase 2 part, two-dose administrations of 3.0 and 5.0 μg ARCT-021 versus placebo separated by 28 days were administered to younger participants (Cohorts F and E) and older participants (Cohorts H and G), respectively. All participants were followed up for 56 days after the last study vaccine administration. Full lists of the inclusion and exclusion criteria are provided in the protocol. Written informed consent was provided by all the participants before enrollment.

Arcturus Therapeutics, Inc. was the regulatory sponsor of the trial. Both Arcturus Therapeutics and Duke-NUS Medical School co-designed the clinical trial and were responsible for the collection, analysis, and interpretation of the data and for the writing of the report. Arcturus Therapeutics and the corresponding author had full access to all the data in the trial and had final responsibility for the decision to submit the manuscript for publication. All the trial data were available to all the authors. Clinical monitoring, pharmacovigilance, and data management were performed by the Contract Research Organization, CTI.

### Trial procedures

Participants were randomized after completing all screening assessments and eligibility criteria. Phase 1 participants were randomized 5:2 to receive ARCT-021 or placebo. Phase 2 participants were randomized 3:1 to receive ARCT-021 or placebo. All participants were administered 0.5 ml injections of ARCT-021 or placebo into the lateral aspect of the deltoid muscle of the non-dominant arm while the second injection (Phase 2 cohorts) was administered into the contralateral arm. All participants were observed for a minimum of 4 h after the injection. Blood samples were obtained for safety and immunogenicity assessments according to protocol schedule. The initial planned single doses to be tested were 1.0, 5.0, 10, and 20 μg in the single-dose cohorts; The final tested doses were 1.0, 5.0, 7.5, and 10 μg in the single-dose cohorts and 3.0 and 5.0 ug in the two-dose cohorts.

### Vaccine and placebo

The ARCT-021 vaccine construct, design and Arcturus Therapeutics proprietary lipid nanoparticle (LNP) has been described in detail in the preclinical publication under LUNAR-COV19 (9). ARCT-021 encodes the native spike protein (GenBank: YP_009724390) of the ancestral strain, along with an alpha-virus replicase complex proteins (specifically the VEEV non-structural proteins nsP1, nsP2, nsP3, and nsP4).

ARCT-021 was presented as a sterile, frozen, aqueous formulation with 0.2 mg/mL of mRNA-2002 and as a 1.0 mL fill (0.2 mg/1 mL) in 2 mL Type I glass vials, stored frozen at −70 °C (±10 °C). It is a white to off-white liquid when thawed with a nominal pH of 8.0 and osmolality of ~1300 mOsm/kg. The placebo was 0.9% sterile saline provided by the study center.

### Safety assessments

Local and systemic solicited and unsolicited AEs were recorded daily by the participants in a symptom diary for at least 7 days and up to 14 days post-vaccination if any events persisted beyond day 7. The symptom diaries were reviewed by site staff at study visits for up to 14 days post each injection. Injection site was inspected at all visits up to day 15 (for first injection)/day 43 (for second injection) or until resolution of local reactogenic event(s). Unsolicited events were collected at all visits and for the duration of study participation.

### Humoral immunity assays

IgM, IgA, and IgG against full-length recombinant S protein were assessed for all cohorts using an in-house Luminex immuno-assay with minor changes to what has been previously published^13^. IgG against the S-protein subdomains (RBD, NTD and S2) were also measured for 5.0 μg, 7.5 μg single and 5.0 μg expansion cohorts. Briefly, his-tagged full-length recombinant S protein or S-protein subdomains (specifically, RBD, NTD and S2) from the ancestral SARC-CoV-2 Wuhan strain expressed in mammalian cells (HEK293) were commercially sourced (Acrobiosystems). Recombinant SARS-CoV-2 protein were directly conjugated to MagPlex-C microspheres (at saturating concentrations of 5 μg/10^6^ beads) and blocked with 1% BSA in PBS. Serum was then serially diluted starting from 1:50 down to a dilution past the endpoint titer, incubated with SARS-CoV-2 protein-conjugated beads for 1 h at 37 °C and using secondary antibodies, probed for IgG (anti-human IgG-PE), IgA (anti-human IgA-biotin and streptavidin-PE) and IgM (anti-human IgM-biotin and streptavidin-PE). Beads were washed in between each experimental step using an automated plate washer, and antibody binding was measured as median fluorescence intensity (MFI) per bead using a Magpix instrument. Measured MFI were then graphed against respective dilution, four parameter logistic (4PL) curves were fitted and the dilution at three-fold over background (i.e., MFI in the absence of serum) were estimated as the serum sample endpoint tier.

Neutralizing antibody was measured using plaque reduction neutralization test (PRNT) with a clinical SARS CoV-2 isolate (hCoV-19/Singapore/2/2020)^13^. Briefly, heat inactivated serum samples were serialy two-fold diluted starting from 1:20, and incubated with virus for 1 h at 37 °C. Antibody-virus mixture were then inoculated onto Vero-E6 cell monolayers in 24-well plates and incubated for 1 h at 37 °C. The antibody-virus mixture was then aspirated and the cells were then overlaid with carboxymethyl cellulose (CMC) with maintenance medium and incubated at 37 °C under 5% CO_2_ for about 4–5 days until viral plaques were formed. Plaques were then counted and the serum titer that neutralizes 50% of the virus inoculum (PRNT50) was calculated.

### Cellular immunity assays

SARS-CoV-2 specific T-cell responses were assessed using intracellular flow cytometry (IFC) and IFNγ ELISPOT assay following stimulation with overlapping S-protein peptide pools^13^. Briefly, IFNγ ELISPOT was conducted by stimulating PBMCs in anti-IFNγ antibody-coated ELISPOT plates with six separate pools of overlapping 15-mer peptides that spanned the full-length SARS-CoV-2 ancestral strain Spike protein (42–43 peptides per pool). Following 18 h of incubation, the ELISPOT plates were sequentially probed with biotinylated anti-IFNγ antibodies and streptavidin-AP, and spot-forming cells developed using KPL BCIP/NBT phosphatase substrate. Spot-forming units (SFU) per well were counted using an automated ELISPOT reader and multiplied by 5 to estimate SFU/million PBMCs.

IFC was conducted on thawed PBMCs, which were incubated for 5 h at 37 °C under 5% CO_2_ with Golgiplug and the 6 separate peptide pools^13^ (13). Using specific anti-human monoclonals antibodies, PBMCs were stained with for the extracellular T-cell surface markers CD3, CD4 and CD8 and the intracellular cytokines IFN-γ, IL-2, TNF-α, and IL-4. Samples were acquired on a BD-LSR II Analyzer and data analyzed using FlowJo software.

### Statistical analysis

No formal sample size calculation was performed. Based on experience from previous studies with other RNA based therapies, the chosen cohort sizes were considered sufficient to meet the objectives of the study while minimizing unnecessary participant exposure. For analysis of safety and humoral immunogenicity, placebo participants were pooled by age group and number of doses administered as follows: A to D1 pooled, E and F pooled, G and H pooled. D2 was the only single dose older adult cohort so D2 placebos were not pooled. For humoral immunogenicity, confidence intervals of the geometric means were calculated with the Student’s *t* distribution on log-transformed data. Seroconversion was defined as at least a four-fold increase in antibody titer from baseline.

### Reporting summary

Further information on research design is available in the Nature Research Reporting Summary linked to this article.

## Supplementary information

Supplementary information

REPORTING SUMMARY

## Data Availability

All study data used to support the findings of this study as well as the clinical trial protocol details, will be available upon request from the corresponding authors.
