# Supplementary material for: A phase I/II randomized, double-blinded, placebo-controlled trial of a self-amplifying Covid-19 mRNA vaccine
Source: NPJ Vaccines. 2022 Dec 13;7:161. doi: 10.1038/s41541-022-00590-x (PMC9745278; doi:10.1038/s41541-022-00590-x)
Supplement: Supplementary file 1 — Supplementary information [file 41541_2022_590_MOESM1_ESM.pdf]

Supplementary Materials

Supplementary Table 1: Summary of Unsolicited Treatment Emergent Adverse Events According to Dose Cohort and Age Group

**Cohort**	**Single Dose**							**Two-Dose**					
	**Age 21 - 55**					**Age 56 - 80**		**Age 21 - 55**			**Age 56 - 80**		
**Event Category / n(%)**	**Cohort A 1µg (N=5)**	**Cohort B 5µg (N=10)**	**Cohort C 10µg (N=5)**	**Cohort D1 7.5µg (N=5)**	**Pooled Placebo Cohorts A to D1 (N=10)**	**Cohort D2 7.5µg (N=5)**	**Cohort D2 Placebo (N=2)**	**Cohort E 5µg (N=12)**	**Cohort F 3µg (N=12)**	**Pooled Placebo Cohorts E and F (N=8)**	**Cohort G 5µg (N=12)**	**Cohort H 3µg (N=12)**	**Pooled Placebo Cohorts G and H (N=8)**
Any Unsolicited Event	4 (80.0)	7 (70.0)	5 (100.0)	4 (80.0)	5 (50.0)	5 (100.0)	0	8 (66.7)	8 (66.7)	6 (75.0)	12 (100.0)	8 (66.7)	3 (37.5)
Related	4 (80.0)	6 (60.0)	5 (100.0)	3 (60.0)	0	5 (100.0)	0	7 (58.3%)	8 (66.7%)	1 (12.5%)	9 (75.0%)	5 (41.7%)	1 (12.5%)
Severe	0	0	2 (40.0)	0	0	0	0	0	0	0	0	1 (8.3)	0
Life threatening	0	0	0	0	0	0	0	0	0	0	0	0	0
Any Serious Unsolicited Event	0	0	0	0	1 (10.0)	0	0	0	0	0	0	0	0
Related	0	0	0	0	0	0	0	0	0	0	0	0	0
Severe	0	0	0	0	0	0	0	0	0	0	0	0	0
Life-threatening	0	0	0	0	0	0	0	0	0	0	0	0	0
Any Unsolicited Event Leading to Withdrawal	0	0	0	0	0	0	0	0	0	0	0	0	0
Related	0	0	0	0	0	0	0	0	0	0	0	0	0
Severe	0	0	0	0	0	0	0	0	0	0	0	0	0
Life-threatening	0	0	0	0	0	0	0	0	0	0	0	0	0
Death	0	0	0	0	0	0	0	0	0	0	0	0	0

Supplementary Table 2: Medically Attended Events by Dose Cohort and Age Group

Cohort	Single Dose							Two-Dose					
	Age 21 - 55					Age 56 - 80		Age 21 - 55			Age 56 - 80		
Event Term / n(%)	Cohort A 1µg (N=5)	Cohort B 5µg (N=10)	Cohort C 10µg (N=5)	Cohort D1 7.5µg (N=5)	Pooled Placebo Cohorts A to D1 (N=10)	Cohort D2 7.5µg (N=5)	Cohort D2 Placebo (N=2)	Cohort E 5µg (N=12)	Cohort F 3µg (N=12)	Pooled Placebo Cohorts E and F (N=8)	Cohort G 5µg (N=12)	Cohort H 3µg (N=12)	Pooled Placebo Cohorts G and H (N=8)
Cellulitis	0	0	0	0	1 (10.0)	0	0	0	0	0	0	0	0
Skin laceration	0	0	0	0	0	0	0	0	1 (8.3)	0	0	0	0
Fever	0	0	0	0	0	0	0	0	0	1 (12.5)	0	0	0
Fatigue	0	0	0	0	0	0	0	0	0	1 (12.5)	0	0	0
Diarrhoea	0	0	0	0	0	0	0	0	0	1 (12.5)	0	0	0
Vomiting	0	0	0	0	0	0	0	0	0	0	1 (8.3)	0	0
Nausea	0	0	0	0	0	0	0	0	0	0	0	1 (8.3)	0
Dizziness	0	0	0	0	0	0	0	0	0	0	1 (8.3)	1 (8.3)	0

Supplementary Table 3: Grade Shifts in Alanine Transaminase and Aspartate Transaminase in ARCT-021 Treated Participants

		ALT					AST				
		Post Vaccination					Post Vaccination				
		**Normal**	**G1**	**G2**	**G3**	**G4**	**Normal**	**G1**	**G2**	**G3**	**G4**
**Baseline**	**Normal**	72 (92.3)	4 (5.1)	0	1 (1.3)	0	75 (96.2)	2 (2.6)	1 (1.3)	0	0
	**G1**	0	1 (1.3)	0	0	0	0	0	0	0	0
	**G2**	0	0	0	0	0	0	0	0	0	0
	**G3**	0	0	0	0	0	0	0	0	0	0
	**G4**	0	0	0	0	0	0	0	0	0	0

Supplementary Table 4: Total Number (%) of Events by Class of Event, Treatment Group and Severity – Safety Population

	Number (%) of Events by Type and Severity							
	ARCT-021-01				Placebo			
**Event Category**	**Grade 1/ Mild**	**Grade 2/ Moderate**	**Grade 3 / Severe**	**Total**	**Grade 1 / Mild**	**Grade 2/ Moderate**	**Grade 3/ Severe**	**Total**
Any Event	387 (78.2)	91 (18.4)	17 (3.4)	495 (100.0)	66 (88.0)	9 (12.0)	0	75 (100.0)
Any Local Solicited Event	142 (28.7)	16 (3.2)	1 (0.2)	159 (32.1)	9 (12.0)	0	0	9 (12.0)
Any Systemic Solicited Event	152 (30.7)	28 (5.7)	11 (2.2)	191 (38.6)	31 (41.3)	4 (5.3)	0	35 (46.7)
Any Unsolicited Adverse Event	93 (18.8)	47 (9.5)	5 (1.0)	145 (29.3)	26 (34.7)	5 (6.7)	0	31 (41.3)

Supplementary Table 5. Antibody responses in 1-Dose Cohorts

	Age 21 – 55										Age 56 – 80			
	1 μg (Cohort A) N=5		5 μg (Cohort B) N=10		7.5 μg (Cohort D1) N=5		10.0 μg (Cohort C) N=5		Pooled Placebo (Cohorts A, B, C, D1) N=10		7.5 μg (Cohort D2) N=5		Cohort D2 Placebo N=2	
**Test/Time point**	**n**	**GMT (95% CI)**	**No**	**GMT (95% CI)**	**n**	**GMT (95% CI)**	**n**	**GMT (95% CI)**	**n**	**GMT (95% CI)**	**n**	**GMT (95% CI)**	**n**	**GMT (95% CI)**
**Luminex anti-S IgG**													2	
Day 1	5	274.0 (111.06, 676.22)	10	166.3 (86.17, 321.01)	5	207.5 (178.89, 240.80)	5	511.7 (61.69, 4244.31)	10	223.7 (126.60, 395.45)	5	141.0 (81.90, 242.68)	2	287.0 (NA)
Day 8	5	364.9 (151.09, 881.32)	10	277.1 (163.72, 468.84)	5	221.0 (145.26, 336.15)	5	995.4 (54.55, 18164.73)	10	278.1 (171.81, 450.11)	5	201.6 (117.94, 344.69)	2	398.1 (NA)
Day 15	5	757.8 (271.89, 2112.02)	10	2217.8 (888.14, 5538.12)	5	3698.4 (2628.51, 5203.68)	5	9482.5 (787.96, 114114.7)	10	267.1 (162.13, 440.09)	5	601.4 (135.44, 2670.21)	2	408.2 (NA)
Day 29	5	2130.0 (516.21, 8788.98)	10	4781.1 (2121.61, 10774.20)	5	13675.7 (6209.51, 30119.08)	5	32748.6 (4963.84, 216057.2)	10	236.5 (133.33, 419.49)	5	2333.3 (265.53, 20502.83)	2	392.7 (NA)
Day 36	5	2542.6 (585.27, 11046.15)	10	4189.3 (2212.27, 7933.24)	5	14898.7 (5117.38, 43375.93)	5	26403.5 (5567.92, 125207.5)	10	289.4 (175.29, 477.68)	5	2119.5 (252.79, 17770.67)	2	330.5 (NA)

	Age 21 – 55										Age 56 – 80			
	**1 µg (Cohort A) N=5**		**5 µg (Cohort B) N=10**		**7.5 µg (Cohort D1) N=5**		**10.0 µg (Cohort C) N=5**		**Pooled Placebo (Cohorts A, B, C, D1) N=10**		**7.5 µg (Cohort D2) N=5**		**Cohort D2 Placebo N=2**	
**Test/Time point**	**n**	**GMT (95% CI)**	**No**	**GMT (95% CI)**	**n**	**GMT (95% CI)**	**n**	**GMT (95% CI)**	**n**	**GMT (95% CI)**	**n**	**GMT (95% CI)**	**n**	**GMT (95% CI)**
Day 43	5	2804.2 (680.03, 11563.45)	10	4958.6 (2484.11, 9898.07)	5	15480.5 (6111.05, 39215.12)	5	28763.3 (4965.70, 166608.3)	10	298.2 (171.99, 517.04)	5	2942.3 (477.76, 18120.22)	2	528.8 (NA)
Day 56	5	2734.4 (616.36, 12130.49)	10	3843.1 (1912.88, 7721.23)	5	13573.3 (4462.61, 41284.14)	5	21768.1 (3916.06, 121002.1)	10	297.2 (151.31, 583.74)	5	2490.5 (318.10, 19498.39)	2	415.0 (NA)
**Live Virus $PRNT_{50}$**														
Day 1	5	20.0 (20.00, 20.00)	10	20.0 (20.00, 20.00)	5	20.0 (20.00, 20.00)	5	20.0 (20.00, 20.00)	10	20.0 (20.00, 20.00)	5	20.0 (20.00, 20.00)	2	20.0 (NA)
Day 29	5	20.0 (20.00, 20.00)	10	24.2 (19.50, 29.95)	5	27.7 (11.21, 68.46)	5	32.9 (14.73, 73.56)	10	20.9 (19.19, 22.68)	5	24.0 (18.61, 30.89)	2	20.0 (NA)
Day 57	5	20.0 (20.00, 20.00)	10	20.0 (20.00, 20.00)	5	20.0 (20.00, 20.00)	5	20.9 (18.48, 23.67)	10	20.0 (20.00, 20.00)	5	20.0 (20.00, 20.00)	2	20.0 (NA)

	Age 21 – 55										Age 56 – 80			
	1 µg (Cohort A) N=5		5 µg (Cohort B) N=10		7.5 µg (Cohort D1) N=5		10.0 µg (Cohort C) N=5		Pooled Placebo (Cohorts A, B, C, D1) N=10		7.5 µg (Cohort D2) N=5		Cohort D2 Placebo N=2	
**Test/Time point**	**n**	**GMT (95% CI)**	**No**	**GMT (95% CI)**	**n**	**GMT (95% CI)**	**n**	**GMT (95% CI)**	**n**	**GMT (95% CI)**	**n**	**GMT (95% CI)**	**n**	**GMT (95% CI)**
**Luminex anti-S IgM**														
Day 1	5	50.0 (50.00, 50.00)	10	73.5 (50.77, 106.51)	5	84.7 (44.63, 160.91)	5	77.9 (34.93, 173.82)	10	67.3 (47.40, 95.49)	5	53.0 (45.06, 62.37)	2	73.1 (NA)
Day 8	5	53.5 (44.37, 64.47)	10	73.6 (53.95, 100.50)	5	63.9 (42.06, 97.20)	5	140.1 (11.23, 1749.25)	10	63.3 (46.52, 86.06)	5	56.1 (40.74, 77.29)	2	116.4 (NA)
Day 15	5	90.6 (28.15, 291.26)	10	427.0 (141.33, 1290.32)	5	215.4 (104.49, 443.91)	5	646.9 (99.38, 4211.00)	10	60.8 (42.94, 86.03)	5	171.1 (69.58, 420.93)	2	136.4 (NA)
**Luminex anti-S IgA**														
Day 1	5	383.1 (36.21, 4052.75)	10	142.1 (56.96, 354.28)	5	101.1 (52.46, 194.97)	5	236.1 (73.94, 754.10)	10	111.7 (86.18, 144.75)	5	61.5 (44.63, 84.76)	2	88.3 (NA)
Day 29	5	2119.1 (159.82, 28096.25)	10	1259.6 (336.74, 4711.46)	5	525.3 (159.82, 1726.38)	5	9056.6 (1083.57, 75696.55)	10	84.9 (64.26, 112.21)	5	321.7 (88.20, 1172.99)	2	162.9 (NA)

	Age 21 – 55										Age 56 – 80			
	1 µg (Cohort A) N=5		5 µg (Cohort B) N=10		7.5 µg (Cohort D1) N=5		10.0 µg (Cohort C) N=5		Pooled Placebo (Cohorts A, B, C, D1) N=10		7.5 µg (Cohort D2) N=5		Cohort D2 Placebo N=2	
**Test/Time point**	**n**	**GMT (95% CI)**	**No**	**GMT (95% CI)**	**n**	**GMT (95% CI)**	**n**	**GMT (95% CI)**	**n**	**GMT (95% CI)**	**n**	**GMT (95% CI)**	**n**	**GMT (95% CI)**
**Luminex anti-RBD IgG***														
Day 1			10	574 (467; 707)	5	631 (411; 969)			6	513 (340; 774)	5	510 (369; 705)	2	926 (8; 113760)
Day 29			10	2618 (1299; 5276)	5	3578 (1171; 10932)			6	653 (389; 1098)	5	1665 (717; 3869)	2	1113 (52; 23697)
**Luminex anti-NTD IgG***														
Day 1			10	276 (242; 314)	5	269 (223; 326)			6	308 (198; 481)	5	269 (202; 357)	2	347 (61; 1964)
Day 29			10	443 (359; 548)	5	446 (260; 766)			6	323 (220; 474)	5	420 (290; 608)	2	309 (174; 549)

	Age 21 – 55										Age 56 – 80			
	1 µg (Cohort A) N=5		5 µg (Cohort B) N=10		7.5 µg (Cohort D1) N=5		10.0 µg (Cohort C) N=5		Pooled Placebo (Cohorts A, B, C, D1) N=10		7.5 µg (Cohort D2) N=5		Cohort D2 Placebo N=2	
**Test/Time point**	**n**	**GMT (95% CI)**	**No**	**GMT (95% CI)**	**n**	**GMT (95% CI)**	**n**	**GMT (95% CI)**	**n**	**GMT (95% CI)**	**n**	**GMT (95% CI)**	**n**	**GMT (95% CI)**
**Luminex anti-S2 IgG***														
Day 1			10	305 (156; 596)	5	204 (149; 279)			6	390 (144; 1055)		192 (116; 317)	2	276 (215; 356)
Day 29			10	9703 (3631; 25928)	5	55676 (18159; 170702)			6	380 (152; 953)		6520 (506; 83940)	2	270 (101; 725)

S = Full-Length SARS-CoV-2 Spike Protein; S2 = S2 domain of SARS-CoV-2 Spike Protein, RBD = receptor binding domain of SARS-CoV-2 Spike Protein; NTD =N-terminal domain of SARS-CoV-2 Spike Protein; PRNT =Plaque Reduction Neutralization Test; GMT=Geometric Mean Titer; CI=Confidence Interval;

N represents the total number of subjects in each cohort. n represents the number of subjects contributing to the summary.

*IgG titers against RBD, NTD and S2 not assessed for subjects in Cohorts A and C.

Supplementary Table 6. Antibody responses in 2-Dose Cohorts

	Age 21 – 55						Age 56 – 80					
**Test/Time point**	**3 µg (Cohort F) N=12**		**5 µg (Cohort E) N=12**		**Cohorts E & F Pooled Placebo N=8**		**3 µg (Cohort H) N=12**		**5 µg (Cohort G) N=12**		**Cohorts G &H Pooled Placebo N=8**	
	**n**	**GMT (95% CI)**	**n**	**GMT (95% CI)**	**n**	**GMT (95% CI)**	**n**	**GMT (95% CI)**	**n**	**GMT (95% CI)**	**n**	**GMT (95% CI)**
**Luminex anti-S IgG**												
Day 1	12	261.9 (170.17, 403.15)	12	321.8 (244.70, 423.10)	8	251.8 (140.56, 451.18)	12	410.2 (221.33, 760.35)	12	334.4 (218.67, 511.27)	8	421.2 (286.14, 620.11)
Day 15	12	1894.6 (511.61, 7016.17)	12	1836.6 (1032.43, 3267.05)	8	201.0 (71.11, 568.04)	12	947.0 (342.52, 2618.17)	12	1423.6 (573.93, 3531.12)	8	339.2 (162.05, 710.14)
Day 29	12	5507.2 (1782.55, 17014.70)	12	5614.9 (3669.54, 8591.57)	8	299.1 (160.18, 558.63)	12	2450.7 (750.65, 8000.85)	12	8407.0 (3437.58, 20560.50)	8	266.2 (135.63, 522.46)
Day 36	12	5677.3 (1683.63, 19144.15)	12	7130.1 (4294.36, 11838.49)	8	215.2 (81.58, 567.53)	12	2971.1 (923.92, 9554.20)	12	7583.3 (3348.57, 17173.44)	8	381.6 (216.40, 672.96)
Day 43	12	14481.0 (6079.57, 34492.51)	12	16771.9 (10507.27, 26771.56)	8	232.4 (109.91, 491.39)	12	5085.5 (1348.87, 19173.26)	12	16513.5 (7432.56, 36689.28)	8	335.8 (128.80, 875.63)

	Age 21 – 55						Age 56 – 80					
**Test/Time point**	**3 µg (Cohort F) N=12**		**5 µg (Cohort E) N=12**		**Cohorts E & F Pooled Placebo N=8**		**3 µg (Cohort H) N=12**		**5 µg (Cohort G) N=12**		**Cohorts G &H Pooled Placebo N=8**	
	**n**	**GMT (95% CI)**	**n**	**GMT (95% CI)**	**n**	**GMT (95% CI)**	**n**	**GMT (95% CI)**	**n**	**GMT (95% CI)**	**n**	**GMT (95% CI)**
Day 57	12	13079.9 (5068.41, 33755.04)	12	16558.1 (9486.89, 28899.99)	8	360.0 (189.44, 684.30)	12	5903.9 (1967.62, 17714.81)	12	10375.4 (5580.18, 19291.17)	8	386.4 (200.55, 744.63)
Day 85	12	9526.0 (4090.22, 22185.61)	12	11592.6 (6630.05, 20269.63)	8	265.9 (113.09, 625.08)	12	5463.5 (2085.67, 14312.11)	12	11998.1 (5247.08, 27435.30)	8	421.1 (205.39, 863.45)
**Live Virus $PRNT_{50}$**												
Day 1	12	20.0 (20.00, 20.00)	12	20.0 (20.00, 20.00)	8	20.0 (20.00, 20.00)	12	20.0 (20.00, 20.00)	12	20.0 (20.00, 20.00)	8	20.0 (20.00, 20.00)
Day 29	12	27.3 17.41, 42.84)	12	29.3 (23.09, 37.13)	8	20.0 (20.00, 20.00)	12	22.5 (19.47, 26.10)	12	20.6 (19.34, 21.88)	8	20.0 (20.00, 20.00)
Day 43	12	34.9 (24.14, 50.38)	12	51.8 (32.30, 83.21)	8	20.0 (20.00, 20.00)	12	27.1 (15.17, 48.35)	12	24.0 (20.56, 27.95)	8	20.0 (20.00, 20.00)

	Age 21 – 55						Age 56 – 80					
**Test/Time point**	**3 µg (Cohort F) N=12**		**5 µg (Cohort E) N=12**		**Cohorts E & F Pooled Placebo N=8**		**3 µg (Cohort H) N=12**		**5 µg (Cohort G) N=12**		**Cohorts G &H Pooled Placebo N=8**	
	**n**	**GMT (95% CI)**	**n**	**GMT (95% CI)**	**n**	**GMT (95% CI)**	**n**	**GMT (95% CI)**	**n**	**GMT (95% CI)**	**n**	**GMT (95% CI)**
Day 57	12	23.8 (17.39, 32.47)	12	36.0 (24.32, 53.16)	8	20.0 (20.00, 20.00)	12	29.7 (15.63, 56.37)	12	20.4 (19.71, 21.09)	8	20.0 (20.00, 20.00)
Day 85	11	24.4 (17.88, 33.30)	12	25.0 (18.94, 33.06)	8	20.0 (20.00, 20.00)	11	27.0 (16.51, 44.13)	12	20.0 (20.00, 20.00)	8	20.0 (20.00, 20.00)
**Luminex anti-S IgM**												
Day 1	12	61.5 (42.99, 88.04)	12	62.6 (50.74, 77.19)	8	71.8 (52.43, 98.23)	12	83.1 (60.16, 114.66)	12	62.7 (47.16, 83.40)	8	59.2 (42.82, 81.82)
Day 15	12	222.7 (108.47, 457.22)	12	194.5 (128.76, 293.83)	8	60.7 (38.50, 95.81)	12	145.1 (64.55, 326.32)	12	114.1 (65.55, 198.53)	8	54.3 (45.75, 64.53)
**Luminex anti-S IgA**												
Day 1	12	149.2 (111.02, 200.55)	12	107.1 (79.89, 143.45)	8	164.9 (119.89, 226.68)	12	182.4 (93.43, 356.08)	12	127.7 (99.85, 163.38)	8	225.2 (157.52, 322.02)

	Age 21 – 55						Age 56 – 80					
**Test/Time point**	**3 µg (Cohort F) N=12**		**5 µg (Cohort E) N=12**		**Cohorts E & F Pooled Placebo N=8**		**3 µg (Cohort H) N=12**		**5 µg (Cohort G) N=12**		**Cohorts G &H Pooled Placebo N=8**	
	**n**	**GMT (95% CI)**	**n**	**GMT (95% CI)**	**n**	**GMT (95% CI)**	**n**	**GMT (95% CI)**	**n**	**GMT (95% CI)**	**n**	**GMT (95% CI)**
Day 29	12	704.6 (445.38, 1114.72)	12	413.6 (255.87, 668.45)	8	186.4 (129.55, 268.28)	12	853.5 (278.06, 2619.52)	12	619.8 (186.82, 2056.61)	8	174.0 (85.99, 352.00)
Day 57	12	519.8 (306.07, 882.76)	12	631.8 (364.71, 1094.58)	8	121.6 (86.83, 170.31)	12	1034.3 (325.44, 3287.32)	12	1174.9 (341.39, 4043.68)	8	250.5 (137.70, 455.66)
**Luminex anti-RBD IgG***												
Day 1			12	648 (480, 876)	4	731 (329, 1624)			12	506 (430, 596)	4	580 (417, 806)
Day 29			12	1606 (1227, 2103)	4	672 (632, 714)			12	1048 (696, 1579)	4	649 (378, 1114)
Day 57			12	4211 (2665, 6656)	4	505 (402, 634)			12	3190 (1744, 5833)	4	798 (553, 1151)

	Age 21 – 55						Age 56 – 80					
**Test/Time point**	**3 µg (Cohort F) N=12**		**5 µg (Cohort E) N=12**		**Cohorts E & F Pooled Placebo N=8**		**3 µg (Cohort H) N=12**		**5 µg (Cohort G) N=12**		**Cohorts G &H Pooled Placebo N=8**	
	**n**	**GMT (95% CI)**	**n**	**GMT (95% CI)**	**n**	**GMT (95% CI)**	**n**	**GMT (95% CI)**	**n**	**GMT (95% CI)**	**n**	**GMT (95% CI)**
**Luminex anti-NTD IgG***												
Day 1			12	466 (296, 731)	4	600 (211, 1709)			12	468 (377, 581)	4	519 (260, 1034)
Day 29			12	862 (746, 995)	4	643 (611, 676)			12	569 (441, 734)	4	567 (353, 913)
Day 57			12	1169 (923, 1479)	4	505 (416, 614)			12	1083 (781, 1502)	4	747 (497, 1124)
**Luminex anti-S2 IgG***												
Day 1			12	590 (334, 1045)	4	1255 (937, 1681)			12	503 (374, 678)	4	486 (234, 1010)

	Age 21 – 55						Age 56 – 80					
**Test/Time point**	**3 µg (Cohort F) N=12**		**5 µg (Cohort E) N=12**		**Cohorts E & F Pooled Placebo N=8**		**3 µg (Cohort H) N=12**		**5 µg (Cohort G) N=12**		**Cohorts G &H Pooled Placebo N=8**	
	**n**	**GMT (95% CI)**	**n**	**GMT (95% CI)**	**n**	**GMT (95% CI)**	**n**	**GMT (95% CI)**	**n**	**GMT (95% CI)**	**n**	**GMT (95% CI)**
Day 29			12	9262 (5455, 15724)	4	1238 (447, 3430)			12	10133 (5034, 20396)	4	546 (410, 726)
Day 57			12	15272 (7489, 31144)	4	1049 (361, 3045)			12	22794 (13235, 39258)	4	798 (675, 944)

S = Full-Length SARS-CoV-2 Spike Protein; S2 = S2 domain of SARS-CoV-2 Spike Protein, RBD = receptor binding domain of SARS-CoV-2 Spike Protein; NTD =N-terminal domain of SARS-CoV-2 Spike Protein; PRNT =Plaque Reduction Neutralization Test; GMT=Geometric Mean Titer; CI=Confidence Interval;

N represents the total number of subjects in each cohort. n represents the number of subjects contributing to the summary.

*IgG titers against RBD, NTD and S2 were not assessed for subjects in cohorts F and H.

Supplementary Table 7. Anti-Spike IgG and Neutralization sero-conversion in 1-Dose Cohorts

	**Age 21 – 55**					**Age 56 – 80**	
**Time point**	**1 µg (Cohort A)**	**5 µg (Cohort B)**	**7.5 µg (Cohort D1)**	**10.0 µg (Cohort C)**	**Pooled Placebo (Cohorts A, B, C, D1)**	**7.5 µg (Cohort D2)**	**Placebo (Cohort D2)**
**Anti-Spike IgG Sero-conversion, % (n/N)***							
Day 1	-	-	-	-	-	-	-
Day 8	0 (0/5)	10 (1/10)	0 (0/5)	20 (1/5)	0 (0/10)	0 (0/5)	0 (0/10)
Day 15	20 (1/5)	90 (9/10)	100 (5/5)	100 (5/5)	0 (0/10)	20 (1/5)	0 (0/10)
Day 29	80 (4/5)	100 (10/10)	100 (5/5)	100 (5/5)	0 (0/10)	80 (4/5)	0 (0/10)
Day 36	80 (4/5)	100 (10/10)	100 (5/5)	100 (5/5)	0 (0/10)	80 (4/5)	0 (0/10)
Day 43	80 (4/5)	100 (10/10)	100 (5/5)	100 (5/5)	0 (0/10)	80 (4/5)	0 (0/10)
Day 57	80 (4/5)	100 (10/10)	100 (5/5)	100 (5/5)	0 (0/10)	80 (4/5)	0 (0/10)
**Live Virus $PRNT_{50}$ Sero-conversion, % (n/N)****							
Day 1	0 (0/5)	0 (0/10)	0 (0/5)	0 (0/5)	0 (0/10)	0 (0/5)	0 (0/10)
Day 29	0 (0/5)	50 (5/10)	20 (1/5)	60 (3/5)	0 (0/10)	80 (4/5)	0 (0/10)
Day 57	0 (0/5)	0 (0/10)	0 (0/5)	20 (1/5)	0 (0/10)	0 (0/5)	0 (0/10)

*Anti-Spike IgG Sero-conversion defined as 4 fold increase in Spike IgG endpoint titer from baseline (i.e. Day 1)

** Anti-Spike IgG Sero-conversion defined as PRNT titers > 20.

Supplementary Table 8. Anti-Spike IgG and Neutralization sero-conversion in 2-Dose Cohorts

	Age 21 – 55			Age 56 – 80		
**Time point**	**3 µg (Cohort F)**	**5 µg (Cohort E)**	**Pooled Placebo (Cohorts E & F)**	**3 µg (Cohort H)**	**5 µg (Cohort G)**	**Pooled Placebo (Cohorts G &H)**
**Anti-Spike IgG Sero-conversion, % (n/N)**						
Day 1	-	-	-	-	-	-
Day 15	75 (9/12)	75 (9/12)	0 (8/8)	17 (2/12)	50 (6/12)	0 (8/8)
Day 29	92 (11/12)	100 (12/12)	0 (8/8)	58 (7/12)	92 (11/12)	0 (8/8)
Day 36	83 (10/12)	100 (12/12)	0 (8/8)	58 (7/12)	92 (11/12)	0 (8/8)
Day 43	100 (12/12)	100 (12/12)	0 (8/8)	75 (9/12)	100 (12/12)	0 (8/8)
Day 57	100 (12/12)	100 (12/12)	0 (8/8)	100 (12/12)	100 (12/12)	0 (8/8)
Day 85	100 (12/12)	100 (12/12)	0 (8/8)	100 (12/12)	100 (12/12)	0 (8/8)
**Live Virus $PRNT_{50}$ Sero-conversion, % (n/N)**						
Day 1	0 (12/12)	0 (12/12)	0 (8/8)	0 (12/12)	0 (12/12)	0 (8/8)
Day 29	25 (3/12)	67 (8/12)	0 (8/8)	25 (3/12)	8 (1/12)	0 (8/8)
Day 43	83 (10/12)	83 (10/12)	0 (8/8)	17 (2/12)	50 (6/12)	0 (8/8)
Day 57	17 (2/12)	58 (7/12)	0 (8/8)	25 (3/12)	17 (2/12)	0 (8/8)
Day 85	17 (2/12)	33 (4/12)	0 (8/8)	17 (2/12)	0 (12/12)	0 (8/8)

*Anti-Spike IgG Sero-conversion defined as 4 fold increase in Spike IgG endpoint titer from baseline (i.e. Day 1)

** Anti-Spike IgG Sero-conversion defined as PRNT titers > 20.

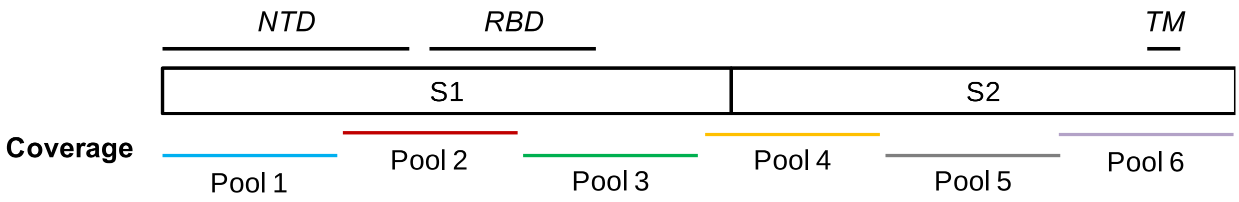

Supplementary Figure 1. Spike location of the peptide pools used in the cellular assays.

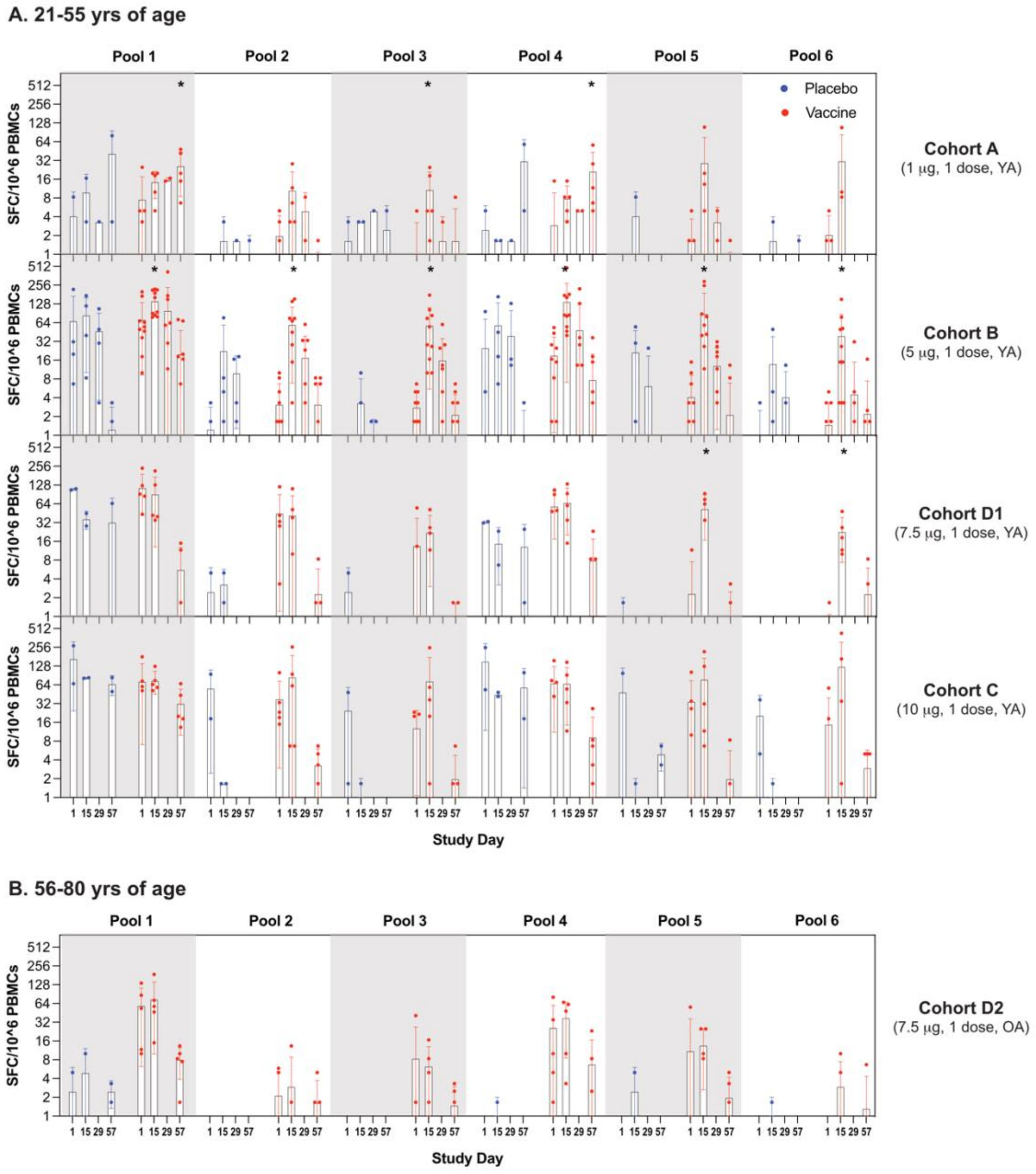

Supplementary Figure 2. T cell responses to a single dose of ARCT-021. T cell responses were measured using an interferon gamma (IFNγ) enzyme-linked immune absorbent spot (ELISPOT) assay and represented as spot-forming cells (SFC) per million PBMCs (Panel A and B). Displayed ELISPOT results are for six peptide pools spanning the entire SARS-CoV-2 Spike. The whiskers display standard deviation (SD) around the mean (horizontal bar). Each dot represents an individual participant. T cell responses at each timepoint were compared to baseline (i.e. day 1) using Mann-Whitney U test. All statistically significant (P<0.05) increases above baseline are marked with an asterisk (*).

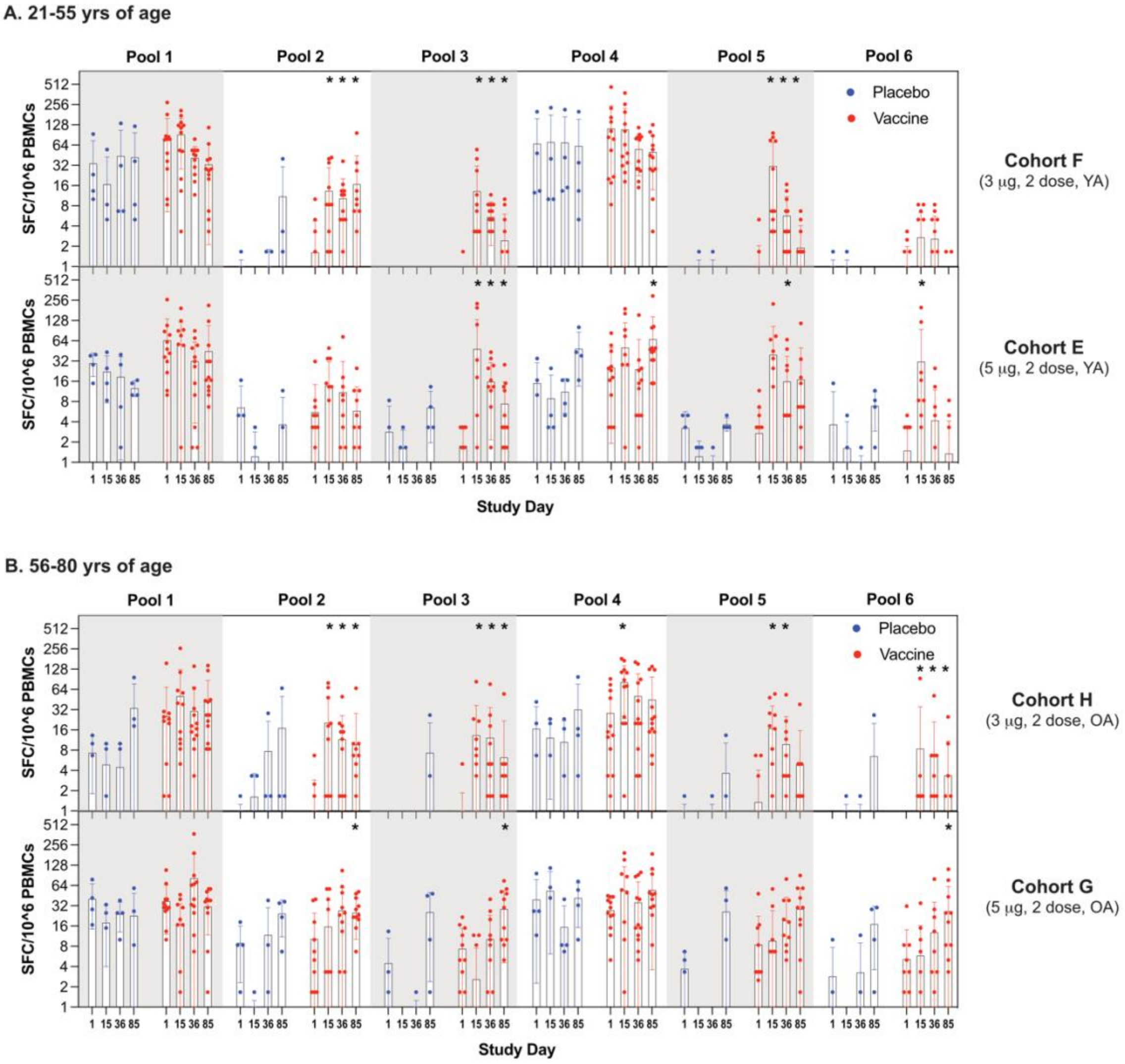

Supplementary Figure 3. Cellular immune responses in 2 dose cohorts. IFNγ ELISPOT responses to six peptide pools spanning the entire SARS-CoV-2 Spike were measured in young (Panel A) and old cohorts (Panel B). ELISPOT responses. In bar graphs, the whiskers display standard deviation (SD) around the mean (horizontal bar). Each dot represents an individual participant. T cell responses at each timepoint were compared to baseline (i.e. day 1) using Mann-Whitney U test. All statistically significant (P<0.05) increases above baseline are marked with an asterisk (*).

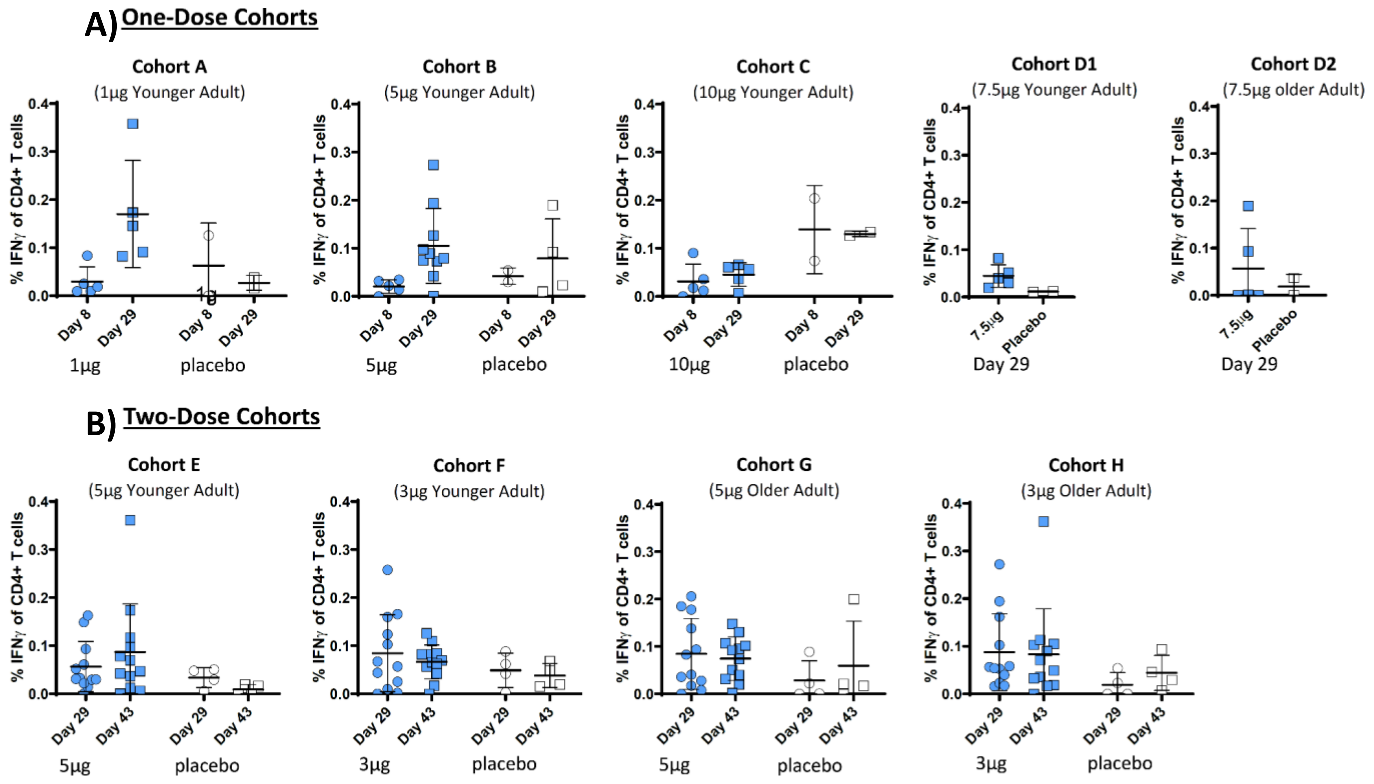

Supplementary Figure 4. Antigen-specific IFNγ CD4 T cell responses by ICS.

Total antigen-specific IFNγ responses in CD4 T cells following stimulation with Spike peptide pools are presented for one-dose (Panel A) and two-dose (Panel B) cohorts. Horizontal bar displays mean per group with error bars depicting the standard deviation (SD). Each dot represents and individual participant. Younger adults 21-55 years; older adults 56-80 years.

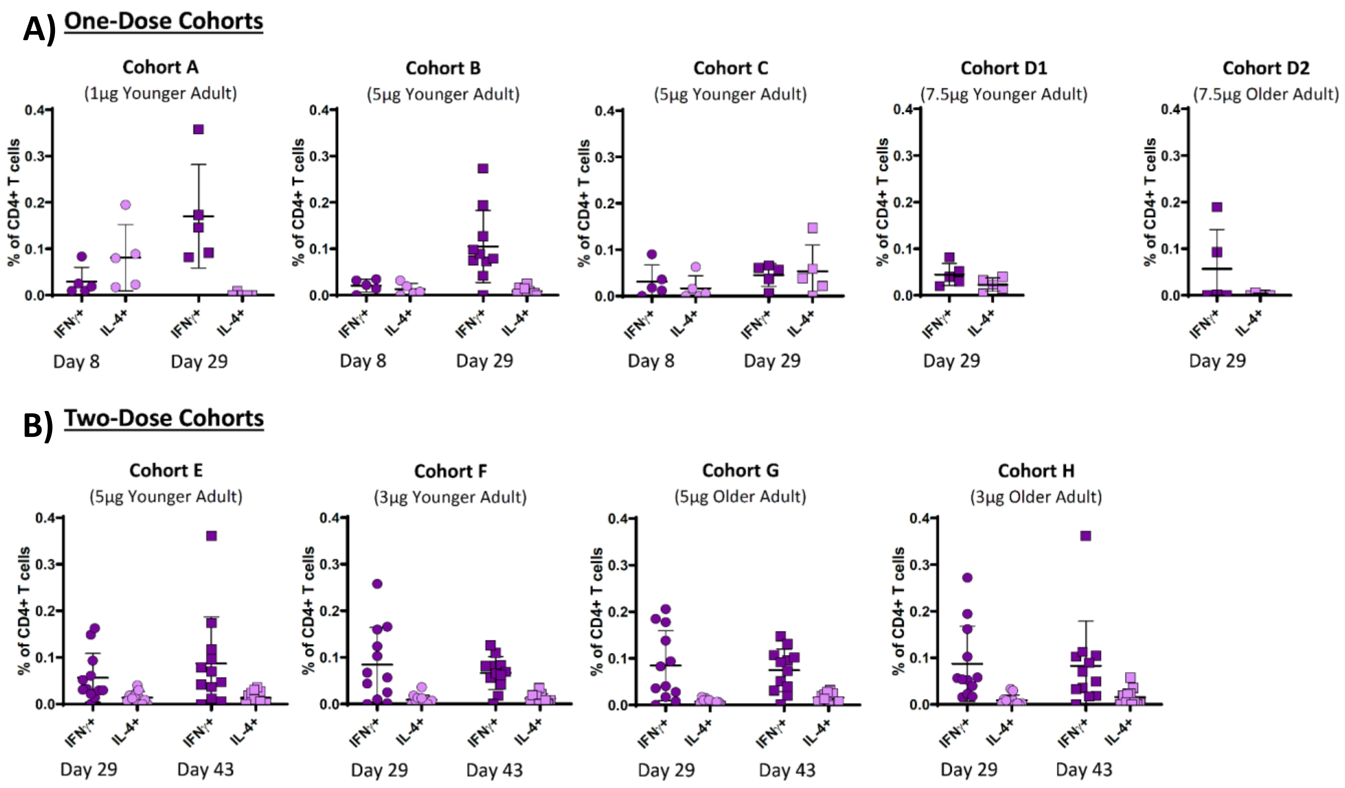

Supplementary Figure 5. Antigen-specific IFNγ and IL4 CD4 T cell responses by ICS.

Total antigen-specific IFNγ and IL4 responses in CD4 T cells following stimulation with Spike peptide pools were measured to assess Th1 responses. Figure presents data for one-dose (Panel A) and two-dose (Panel B) cohorts. Horizontal bar displays mean per group with error bars depicting the standard deviation (SD). Each dot represents and individual participant. Younger adults 21-55 years; older adults 56-80.

No notable differences between ARCT-021 and placebo for Il-2 and TNF-alpha responses and data therefore not shown.

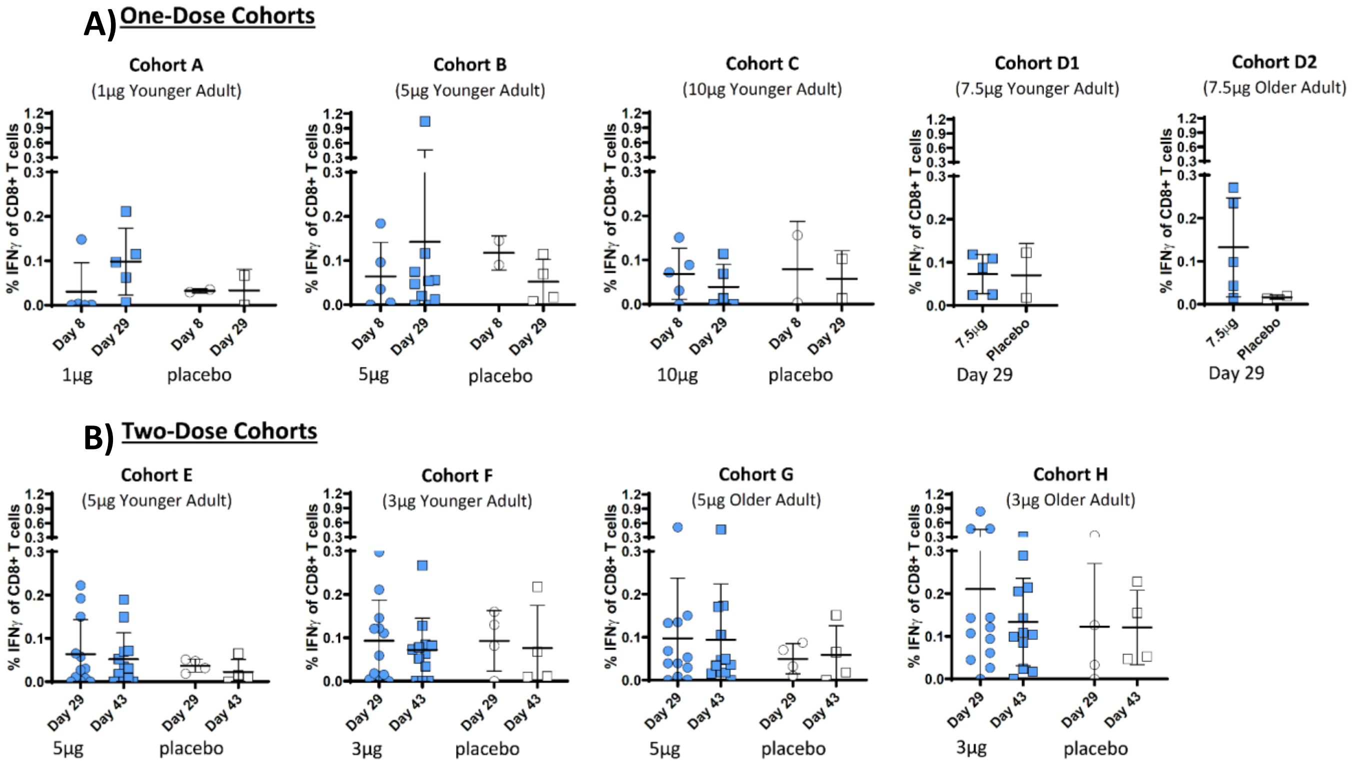

Supplementary Figure 6. Antigen-specific IFNγ CD8 T cell responses by ICS.

Total antigen-specific IFNγ responses in CD8 T cells following stimulation with Spike peptide pools are presented for one-dose (Panel A) and two-dose (Panel B) cohorts. Horizontal bar displays mean per group with error bars depicting the standard deviation (SD). Each dot represents and individual participant. Younger adults 21-55 years; older adults 56-80 years.

No notable differences between ARCT-021 and placebo for Il-2 and TNF-alpha responses and data therefore not shown.
