# Supplementary material for: A phase I/II randomized, double-blinded, placebo-controlled trial of a self-amplifying Covid-19 mRNA vaccine
Source: NPJ Vaccines. 2022 Dec 13;7:161. doi: 10.1038/s41541-022-00590-x (PMC9745278; doi:10.1038/s41541-022-00590-x)
Supplement: Supplementary file 2 — REPORTING SUMMARY [file 41541_2022_590_MOESM2_ESM.pdf]

nature portfolio

Corresponding author(s): Professor Eng Eong Ooi

Last updated by author(s): Nov 23, 2022

Reporting Summary

Nature Portfolio wishes to improve the reproducibility of the work that we publish. This form provides structure for consistency and transparency in reporting. For further information on Nature Portfolio policies, see our Editorial Policies and the Editorial Policy Checklist.

Statistics

For all statistical analyses, confirm that the following items are present in the figure legend, table legend, main text, or Methods section.

n/a	Confirmed	
☐	☒	The exact sample size (*n*) for each experimental group/condition, given as a discrete number and unit of measurement
☒	☐	A statement on whether measurements were taken from distinct samples or whether the same sample was measured repeatedly
☐	☒	The statistical test(s) used AND whether they are one- or two-sided<br>*Only common tests should be described solely by name; describe more complex techniques in the Methods section.*
☐	☒	A description of all covariates tested
☐	☒	A description of any assumptions or corrections, such as tests of normality and adjustment for multiple comparisons
☐	☒	A full description of the statistical parameters including central tendency (e.g. means) or other basic estimates (e.g. regression coefficient) AND variation (e.g. standard deviation) or associated estimates of uncertainty (e.g. confidence intervals)
☐	☒	For null hypothesis testing, the test statistic (e.g. *F*, *t*, *r*) with confidence intervals, effect sizes, degrees of freedom and *P* value noted<br>*Give P values as exact values whenever suitable.*
☒	☐	For Bayesian analysis, information on the choice of priors and Markov chain Monte Carlo settings
☒	☐	For hierarchical and complex designs, identification of the appropriate level for tests and full reporting of outcomes
☒	☐	Estimates of effect sizes (e.g. Cohen's *d*, Pearson's *r*), indicating how they were calculated

Our web collection on statistics for biologists contains articles on many of the points above.

Software and code

Policy information about availability of computer code

Data collection	All clinical trial data was collected by a contract research organization called CTI, using electronic data collection methods such as imedidata. On-site and remote monitoring of sites by Sponsor (or designee) with 100% source data verification to ensure compliance with protocol and accuracy of data collection/transcription.
Data analysis	All data analysis was conducted using GraphPad Prism software version 9.

For manuscripts utilizing custom algorithms or software that are central to the research but not yet described in published literature, software must be made available to editors and reviewers. We strongly encourage code deposition in a community repository (e.g. GitHub). See the Nature Portfolio guidelines for submitting code & software for further information.

Data

Policy information about availability of data

All manuscripts must include a data availability statement. This statement should provide the following information, where applicable:

- Accession codes, unique identifiers, or web links for publicly available datasets
- A description of any restrictions on data availability
- For clinical datasets or third party data, please ensure that the statement adheres to our policy

All study data used to support the findings of this study as well as the clinical trial protocol details, will be available upon request from the corresponding authors.

Human research participants

Policy information about studies involving human research participants and Sex and Gender in Research.

Reporting on sex and gender	Sex information of participants has been collected and presented in table 1.
Population characteristics	Population characteristics such as sex, race and age distribution of the study participants are presented in table 1.
Recruitment	Following consent, participants were randomized into treatment groups. Both participants and investigator were blinded to the treatment group - therefore this was a double blind study.
Ethics oversight	The trial was conducted at the Singapore Health Services (SingHealth) Investigational Medicine Unit, following approvals by the SingHealth Centralized Institutional Review Board (CIRB F/2020/2553) and the Singapore Health Sciences Authority.

Note that full information on the approval of the study protocol must also be provided in the manuscript.

Field-specific reporting

Please select the one below that is the best fit for your research. If you are not sure, read the appropriate sections before making your selection.

☒ Life sciences ☐ Behavioural & social sciences ☐ Ecological, evolutionary & environmental sciences

For a reference copy of the document with all sections, see nature.com/documents/nr-reporting-summary-flat.pdf

Life sciences study design

All studies must disclose on these points even when the disclosure is negative.

Sample size	No formal sample size calculation was performed. Based on experience from previous studies with other RNA based therapies, the chosen cohort sizes are considered sufficient to meet the objectives of the study while minimizing unnecessary subject exposure; specifically 5 subjects exposed to ARCT-021 at each dose level is sufficient to determine which doses should be evaluated further and 12 subjects exposed to ARCT-021 in each expansion cohort is sufficient to select the optimal dose for subsequent clinical trials. For analyses, control subjects were pooled and analysed as separate control groups.
Data exclusions	No data was excluded in the analysis
Replication	All experimental studies on clinical trial samples were conducted in duplicates or triplicates, as well as in-built experiment controls to minimize experimental error.
Randomization	Subjects were randomized after all Screening assessments have been completed and after the Investigator has verified that they are eligible per inclusion and exclusion criteria. No subject began study treatment prior to randomization and assignment of a unique subject identification number. Within dose escalation cohorts A to C, subjects were randomized 5:2 to receive ARCT-021 or placebo. Within expansion cohorts D and E and older adult cohorts F and G, subjects were randomized 3:1 to receive ARCT-021 or placebo. The Sponsor or designee prepared the randomization list, which were provided to the study site unblinded pharmacist.
Blinding	The Sponsor and all subjects, monitors, and Study Center personnel related to the study were blinded throughout the study, except for the pharmacist (or qualified designee) who dispenses/prepares the Study Drug (ARCT-021 or placebo) and the pharmacy monitor who monitors the pharmacy records and procedures.

Reporting for specific materials, systems and methods

We require information from authors about some types of materials, experimental systems and methods used in many studies. Here, indicate whether each material, system or method listed is relevant to your study. If you are not sure if a list item applies to your research, read the appropriate section before selecting a response.

Materials & experimental systems

n/a	Involved in the study
☐	☒ Antibodies
☐	☒ Eukaryotic cell lines
☒	☐ Palaeontology and archaeology
☒	☐ Animals and other organisms
☐	☒ Clinical data
☒	☐ Dual use research of concern

Methods

n/a	Involved in the study
☒	☐ ChIP-seq
☒	☐ Flow cytometry
☒	☐ MRI-based neuroimaging

Antibodies

Antibodies used	PE conjugated Goat anti-Human IgG Fc Secondary Antibody (eBioscience Cat#12-4998-82)<br>Goat Anti-Human IgM-biotin (SouthernBiotech Cat#2020-08)<br>Goat Anti-Human IgA-biotin (SouthernBiotech Cat#2050-08)<br>Streptavidin-PE (SouthernBiotech Cat#7105-09L)
Validation	Antibodies validated by manufacturer

Eukaryotic cell lines

Policy information about cell lines and Sex and Gender in Research

Cell line source(s)	VERO C1008 (Vero 76, clone E6, Vero E6 - CRL-1586) were originally purchased from ATCC
Authentication	Vero-E6 cell line authenticated by by ATCC prior to purchase.
Mycoplasma contamination	Vero-E6 regularly tested for mycoplasma contamination. Cells used for current study were mycoplasma-free.
Commonly misidentified lines (See ICLAC register)	not applicable

Clinical data

Policy information about clinical studies

All manuscripts should comply with the ICMJE guidelines for publication of clinical research and a completed CONSORT checklist must be included with all submissions.

Clinical trial registration	NCT04480957
Study protocol	Study protocol is available upon request.
Data collection	Randomized, double blinded (study site staff, subjects and Sponsor), placebo controlled, adaptive, ascending dose study evaluating administration of ARCT-021 in healthy adult subjects in Singapore. 0.9% sterile saline will serve as a placebo control. Study drug (ARCT-021 or control) will be administered as an intramuscular (IM) injection. The study comprises two parts. In the Phase 1 part escalating dose levels given as a single injection to younger adults (21 to 55 years) will be evaluated sequentially. Two dose levels will be further evaluated in the Phase 2 part of the study in two expansion cohorts in younger adults (21 to 55 years) and in two elderly subject (56 to 80 years) cohorts.
Outcomes	Primary Outcome Measures :<br>Incidence, severity and dose-relationship of AEs [Time Frame: 56 days]<br>Safety and tolerability of ARCT-021 assessed by determining the incidence, severity and dose-relationship of AEs by dose<br><br>Secondary Outcome Measures :<br>Geometric mean titre for SARS-CoV-2-specific serum neutralizing antibody [Time Frame: Up to 56 days]<br>SARS-CoV-2-specific serum neutralizing antibody levels, expressed as GMT<br><br>Mean titre for SARS-CoV-2-specific serum neutralizing antibody levels [Time Frame: Up to 56 days]<br>SARS-CoV-2-specific serum neutralizing antibody levels, expressed as mean titer<br><br>Geometric mean fold rise in titre for SARS-CoV-2-spike protein specific neutralizing antibody levels [Time Frame: Up to 56 days]<br>GMFR in titre for SARS-CoV-2-spike protein specific neutralizing antibodies from before vaccination to each subsequent time point<br><br>Other Outcome Measures:<br>Increase in SARS-CoV-2--spike protein-specific binding antibody levels [Time Frame: Up to 56 days]<br>GMFR in SARS-CoV-2--spike protein-specific binding antibody levels from before vaccination to each subsequent time point

Geometric mean SARS-CoV-2--spike protein-specific binding antibody titre [Time Frame: Up to 56 days]
GMT for SARS-CoV-2--spike protein-specific binding antibody levels

Mean SARS-CoV-2--spike protein-specific binding antibody titre [Time Frame: Up to 56 days]
Mean titer for SARS-CoV-2--spike protein-specific binding antibody levels

SARS-CoV-2-specific serum neutralizing antibody seroconversion rate [Time Frame: 56 days]
Proportion of participants that are seronegative before vaccination achieving a titer of greater than or equal to 20 for SARS-CoV-2-specific serum neutralizing antibodies

SARS-CoV-2-specific serum neutralizing antibody seroconversion rate (seropositive baseline) [Time Frame: 56 days]
Proportion of participants that are seropositive before vaccination achieving a greater than or equal to 4-fold rise from before vaccination in SARS-CoV-2-specific serum neutralizing antibody levels
